# Impact of Hop Freshness on Dry Hopped Beer Quality

**DOI:** 10.3390/foods11091310

**Published:** 2022-04-30

**Authors:** Ksenija Rutnik, Miha Ocvirk, Iztok Jože Košir

**Affiliations:** Department for Agrochemistry and Brewing, Slovenian Institute of Hop Research and Brewing, 3310 Žalec, Slovenia; ksenija.rutnik@ihps.si (K.R.); miha.ocvirk@ihps.si (M.O.)

**Keywords:** aged hops, beer quality, hop storage index

## Abstract

The hop plant is seasonal, but beer production continues throughout the whole year. The quality of hops begins to decrease immediately after harvesting; therefore, maintaining the highest possible quality is important. A good indicator of hop freshness is the hop storage index (HSI). In this study, three different varieties of hops with five different HSI values, from 0.3 to 0.7, were used for brewing with the dry hopping technique. The main goal was to evaluate the impact of the HSI value on beer quality in terms of hop aroma and bitterness. Alpha acids, iso-alpha acids, humulinones, bitterness units and hop aroma compounds were chemically analysed. Sensorial analysis was also conducted on all samples. Decreases in the intensity and quality of hop aroma were detected with increasing HSI. The quality of bitterness was also reduced. High HSI also led to undesirable gushing. Beers brewed with hops with HSI values greater than 0.4 had deviations in aroma and bitterness when compared with beers brewed with fresh hops.

## 1. Introduction

Dry hopping has become an indispensable technique in beer production, especially in craft breweries. In this process, hops are added to cooled down wort, during fermentation or maturation, mainly to impart the aroma and flavour of beer [1]. Technically, dry hopping is the cold extraction of hop volatiles and non-volatiles. When traditional kettle hopping is applied, most of the volatiles evaporate during boiling and the contribution to beer aroma is limited. In dry hopping, there is no boiling, so the direct transfer of hop constituents is allowed and, consequentially, higher amounts are present in beer. Therefore, dry hopped beers have a more intense aroma, while bitterness is reduced compared to kettle hopped beers [2]. However, there are numerous variables that have to be taken into account when dry hopping is applied. Its efficiency is dependent on the number of hop additions, contact time, temperature, the regime (static or dynamic) and, last but not least, hop parameters such as alpha acid content, essential oils, form of hops, polyphenols, etc. [3].

For a long time, dry hopping was not considered to contribute to bitterness, since no isomerisation of alpha acids occurs. However, this idea has been revisited recently by researchers who have investigated the influence of dry hopping on beer bitterness [4,5,6]. For example, Parkin and Shellhammer [4] reported that bitterness in dry hopped beers is also derived from humulinones and polyphenols. However, the majority of the contribution comes from the former, since polyphenols have a tenfold lower influence on bitterness units (BUs) and perceived bitterness.

Humulinones are oxygenated products of alpha acids, and their levels increase during hop storage. They are approximately 66% as bitter as iso-alpha acids; therefore, they should be desirable components in hops to impart bitterness during dry hopping [7,8,9]. However, during the period when the levels of humulinones are increasing, numerous other changes are also occurring in the chemical composition of hops. One is that the levels of hop essential oil decrease and its composition changes significantly during storage. The oxygenated fraction of hop oil increases at the expense of hydrocarbons, and various compounds are interconverted. Therefore, the aroma imparted by old hops could be completely different from that typically attained with fresh hops [9].

The parameter most widely used for determining the freshness of hops is the hop storage index (HSI). The HSI value is used to sort hops into five categories of freshness: fresh, slightly aged, aged, strongly aged and overaged [10]. Many brewers have strict requirements about the upper limit of HSI, although, to our knowledge, only two studies evaluating hop HSI effects on beer bitterness and aroma have been published [11,12]. However, these studies only compared beers brewed with fresh and aged hops, and kettle hopping was applied. Our goal in the present study was to expand the knowledge in this field by determining the HSI values when aged hops perceptually influence beer aroma. The research was conducted using hops of three different Slovenian varieties at HSI values of 0.3, 0.4, 0.5, 0.6 and 0.7. The BUs of alpha acids, iso-alpha acids, humulinones and hop essential oil compounds were also measured in beer samples, and sensorial analysis was performed.

## 2. Materials and Methods

### 2.1. Chemicals and Standards

Methanol (HPLC grade) was purchased from J. T. Baker (Deventer, The Netherlands); toluene, phosphoric acid (85%) and hydrochloric acid (37%) were obtained from Honeywell (Charlotte, NC, USA); 2, 2, 2-trimethyl pentane (≥99%, ACS reagent), 1-butanol, sodium chloride, sodium hydroxide, ammonium iron (III) citrate, carboxymethyl cellulose (CMC), alpha-pinene (98%), beta-pinene (99%), myrcene (99%), limonene (99%), linalool (97%), alpha-terpineol (99%), beta-cutronellol (99%), nerol (98%), geraniol (98%), geranyl acetate (98%), beta-caryophyllene (99%), alpha-humulene (CRM) and caryophyllene oxide (95%) were purchased from Sigma Aldrich (St. Louis, MO, USA); ethylenediaminetetraacetic acid (EDTA) was obtained from Merck (Kenilworth, NJ, USA); international calibration extract 4 (ICE4), DCHA-Iso and DCHA-Humulinones were obtained from Labort Veritas (Zürich, Switzerland). Light Malt Extract was procured from Muntons (Lombard, Chicago, IL, USA).

### 2.2. Hops Material

Three different varieties (Celeia, Aurora and Styrian Wolf) were supplied by Hmezad Exim d.d. (Žalec, Slovenia). The HSI was immediately measured, and the first three samples (one from each variety) were stored at −18 °C. The rest of the hops were exposed to ambient air and temperature, and HSI was monitored weekly. Additional samplings were made for each variety when the HSI value increased to 0.4, 0.5, 0.6 and 0.7. Altogether, 15 hop samples were stored in the freezer until used in the experiment.

### 2.3. Dry Hopping

Wort was prepared by mixing six kilograms of British Light Extract (Muntons Malted Ingridients, Chicago, IL, USA), 56 L of water and 3.36 kg of dextrose for 1 h. Analytical specifications for British Light Extract obtained by the producer were as follows: refractometric solids were 79.5 to 82%, pH was between 5 and 6, and the colour EBC (10% *w/v* solution) was lower than 10. The mixture was placed into a fermentation tank and 1 g/L of yeast (SafAle^TM^ S-04) was added. Fermentation proceeded for 7 days. The desired hop sample (10 g/L) was added to fermented wort and left for 5 days at 20 °C. After 5 days, the beer was filtered through gauze to remove the hops and samples were taken for chemical analysis and stored at −18 °C until use. For sensory analysis, the beers were poured into bottles and kept in a cold room (4 °C) until sensory evaluation. All experiments were conducted in duplicate.

### 2.4. Sensory Analysis

Sensory analysis was conducted by a panel of ten assessors (6 males, 4 females, aged 26–55 years), trained in FlavorActiV sensory training. A routine descriptive test was chosen for sensory analysis, following method 13.13 in Analytica-EBC [13]. The panellists were asked to assess the intensity and the quality of hop aroma and bitterness of 15 beer samples. The overall impression was also one of the attributes. The scale for all attributes ranged from 1 to 5, with half-point steps allowed. All samples were blind-labelled and presented to the assessors in three series of five samples, each set presenting its own hop variety with different HSI values. Seventy millilitres of each sample were served, tempered to 10 °C, in a 250 mL glass. No discussion was permitted until the answer forms were handed in. One-way ANOVA, followed by a Tukey test (α = 0.05), was performed for statistical processing of the data. Data were analysed using the OriginPro^®^ 2020b (OriginLab Corporation, Northampton, MA, USA) software package.

### 2.5. Chemical Analysis

#### 2.5.1. Hop Storage Index

The HSI value was determined according to the Analytica-EBC method [14]. In short, 25 mL of toluene was added to ground hops (2.5 g) and placed in a shaker for 45 min. After extraction, the hops were removed by filtering the extract. Two millilitres of the filtered solution was diluted to 50 mL with methanol (solution A). Solution A was further diluted with alkaline methanol in a 1:24 ratio (v/v) to produce solution B. The absorbance of solution B was measured spectrophotometrically against the blank sample at 275 nm and 325 nm. The ratio between these wavelengths gave the HSI value [14].

#### 2.5.2. Bitterness Units (BUs) of Beer

The BUs of the beers were measured following the official method prescribed by Analytica-EBC [15]. Briefly, 0.5 mL of hydrochloric acid and 20 mL of iso-octane were added to 10 mL of degassed beer. The mixture was shaken for 15 min and centrifuged for 3 min (3000 rpm). The absorbance of the iso-octane layer was measured spectrophotometrically at 275 nm against pure iso-octane. The BU was calculated according to Equation (1):

BU = 50 · A_275_ (1)

where

A_275_ is the absorbance at 275 nm measured against a reference of pure iso-octane.

The results were rounded to the nearest whole number.

#### 2.5.3. HPLC Analysis of Alpha Acids, Iso-Alpha Acids and Humulinones

Beer samples were shaken to degas the carbon dioxide. Before analysis, the samples were centrifuged at 3000 rpm for 15 min and filtered into vials through 0.45 μm PTFE filters. The components were separated on a Nucleodur^®^ 5–100 C18, 125 × 4 mm (Macherey-Nagel, Düren, Germany) HPLC column. Distilled water, methanol and orthophosphoric acid, in a ratio of 775:210:9 (v/v/v), was used as the isocratic mobile phase. The thermostat was set at 40 °C and the flow rate at 1 mL/min, and 2 μL of sample was injected. The eluted compounds were detected with a diode array detector (DAD) set at 314 nm for alpha acids and 270 nm for iso-alpha acids and humulinones. The external standards were ICE4, DCHA-Iso and DCHA-humulinones. HPLC analysis was performed with an Agilent 1200 Series (Agilent, Santa Clara, CA, USA) chromatography system, and the data handling was done using Agilent ChemStation for LC 3D systems (Rev. B.03.02) [16].

#### 2.5.4. HS-SPME-GC-MS Analysis of Aroma Components Derived from Hops

Beer samples (10 mL) were transferred into 20 mL vials and 1 g of sodium chloride and 0.5 mL of internal standard iso-butanol were added. The samples were analysed by gas chromatography (Agilent 8890 GC System; Agilent, USA) coupled with mass spectrometry (597BB GC/MS; Agilent, USA) using a system equipped with an autosampler (PAL RSI 120). Agilent MSD ChemStation Enhanced Data Analysis (Rev. F.01.036.2357) was used for data analysis. The parameters and operating conditions are listed in Table 1, following the method described by Dennenlöhr et al. [17].

Calibration curves with eight calibration points were constructed for myrcene, linalool, alpha-terpineol, beta-citronellol, nerol, geraniol, geranyl acetate, beta-caryophyllene, alpha-humulene and caryophyllene oxide. The obtained correlation coefficients were more than 0.99 and the relative standard deviations (RSDs) were up to ±16%.

## 3. Results and Discussion

When hops are harvested, their chemical composition starts to change immediately. Proper storage conditions can slow these changes down; however, they are inevitable. Since hops are seasonal plants, continuous use of fresh hops is not possible. One of the measures of hop freshness is the HSI. Therefore, our main goal was to determine the HSI value at which hops are no longer suitable for dry hopping. The dry hopping process uses large quantities of hops per L of wort; therefore, any storage changes could have negative effects on the beer aroma profile.

### 3.1. Hops Analysis

All hop samples, before starting the experiment, were analysed for alpha acid, humulinone and hop essential oil content (Table 2).

The essential oil content and the levels of alpha acids dropped with increasing HSI values in all three varieties. The levels of humulinones increased, as expected, since humulinones are oxidation products of alpha acids. Standard deviations for essential oil, alpha acids and humulinones are presented in Table 2 together with the results, while the standard deviation for HSI is not presented, since it is 0.01 in all cases.

### 3.2. Beer Aroma

Table 3 shows the levels of 11 hop essential oil compounds in the beer samples. Myrcene, alpha-humulene and beta-caryophyllene have low solubility in water, but their levels were among the highest due to their high concentration in hop oil. Myrcene is one of the most potent odourants in beer, since it has the lowest detection threshold (9.5 ppb) among the main hydrocarbons [18]. In dry hopped beers, these levels are usually higher, which results in the traditional hoppy aroma of beer. Levels of myrcene were higher than the threshold in all the samples. However, the concentration of myrcene was up to elevenfold lower in the beers dry hopped with hops with HSI values of 0.7.

Alpha-humulene has a significantly higher threshold than myrcene, at around 450 ppb [19]. Alpha-humulene imparts a spicy and woody character to beer [18]. Only one beer sample, dry hopped with Celeia (HSI 0.3), reached the threshold value. The levels in Aurora beer (HSI 0.3) were slightly below the threshold value, whereas all other samples showed lower levels, indicating only a limited contribution to the overall aroma.

Beta-caryophyllene exhibits the same characteristics as alpha-humulene in terms of odour description and threshold values [19,20]. The levels of beta-caryophyllene were higher overall in our samples when compared to alpha-humulene. Nevertheless, the levels were still below the threshold values, with the exception of beer brewed/dry hopped with Celeia and beer brewed/dry hopped with Aurora (both with HSI 0.3).

One of the most significant contributors to the overall aroma of beer is definitely linalool. It has a low threshold (27 ppb), which is almost certainly achieved when the dry hopping technique is applied [18,21]. Linalool also imparts a pleasant floral aroma and is one of the most desired hop oil compounds in beer. The greatest decrease in linalool occurred when Aurora was used for brewing. However, the levels were still at least 10 times higher than the threshold values.

Besides linalool, four other monoterpene alcohols contribute to beer aroma. Monoterpene alcohols impart floral and citrus characters to beer, although nerol and alpha-terpineol, due to their higher threshold values, are detected at lower levels when compared to linalool, geraniol and beta-citronellol [22]. Geraniol adds fruity and floral notes and is labelled as one of the most potent odourants contributing to beer aroma [23]. The concentrations of geraniol were lowered, resulting in a lower intensity and quality of the aroma. The concentrations of beta-citronellol and nerol were combined due to insufficient peak resolution. Their levels did not follow a strict pattern, probably because of the many conversions among monoterpene alcohols occurring during dry hopping. Linalool can arise from nerol or geraniol, and beta-citronellol can be produced from geraniol. Linalool could also be cyclised to alpha-terpineol [24]. Alpha-terpineol levels were far below the threshold values, so their contribution to aroma in our samples is negligible.

The oxidation products beta-caryophyllene oxide, humulene epoxide and humulenol II were detected. Humulene epoxide and humulenol concentrations were measured as relative percentages, since no standards were available. In contrast to other compounds, their levels increased. These compounds are known to impart a musty and mouldy character to beer [25]. In general, the greatest decrease in aroma compounds was observed between HSI 0.3 and 0.4, regardless of the variety used for dry hopping. Increasing HSI was associated with a general decrease in the concentrations of key hop compounds, resulting in a less intense aroma and reduced beer quality.

However, when investigating hop aroma, a wider perspective should be applied, since some compounds, when present together, can have additive, synergistic, antagonistic or eliminating effects. For this reason, sensorial analysis was performed. The results are summarised in Table 4. Results are given as the mean value ± standard deviation from ten independent evaluators. The highest intensity and quality of hop aroma was determined for Styrian Wolf with HSI 0.3. In all varieties, both the intensity and quality declined with increasing HSI. Beers dry hopped with the oldest hops were marked as the worst in terms of the intensity and quality of beer aroma.

**Figure 1:**
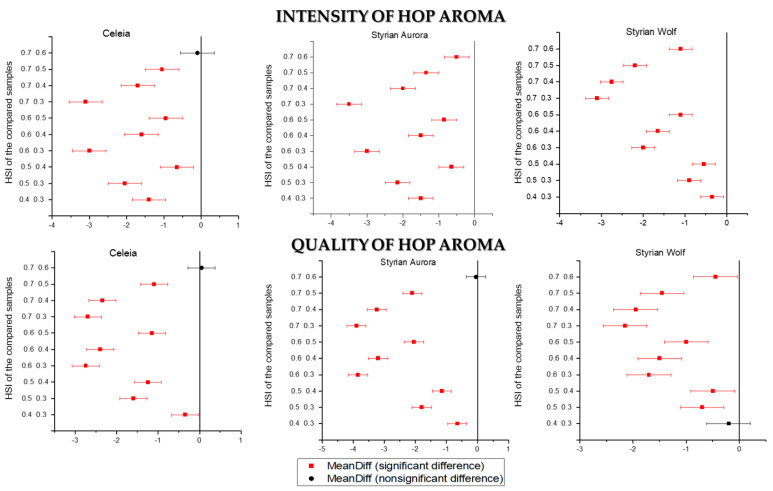
Tukey’s mean comparison plots for intensity and quality of hop aroma.

Figure 1 shows the Tukey mean comparison plots. On the x-axis, the mean difference between samples is presented, and on the y-axis, the HSIs of the compared samples are presented. The level of confidence α is 0.05. Observing the results of the Tukey test for the intensity of hop aroma in Celeia, it is seen that only when samples with HSI 0.6 and 0.7 (the highest row on y-axis) are compared, there is no significant difference (black dot) between them at the 0.05 level. All other samples are statistically different (red squares) at the 0.05 level. The sum of the most contributory compounds was lowest among beers dry hopped with Styrian Wolf hops, and yet the sensorial intensity was evaluated as the strongest. This could be a consequence of the higher levels of geraniol and linalool present in the Styrian Wolf samples. For Celeia, the greatest decrease in the intensity of the hop aroma was observed between HSI 0.3 and 0.4. In terms of quality, this drop appeared one step later, between HSI 0.4 and 0.5. After HSI 0.6, the samples were no longer statistically different, as the intensity of the hop aroma was low and the aroma was no longer pleasant. For Aurora, the greatest change in intensity and quality was between HSI 0.5 and 0.6. The samples differed statistically in the intensity of the aroma and in the quality of the aroma, except for samples 9 and 10. After HSI 0.5, the intensity of the hop aroma was low and it became unpleasant. Therefore, hops of the Aurora variety with an HSI higher than 0.5 are definitely unsuitable for dry hopping. An interesting pattern was observed with the Styrian Wolf variety, as even hops with HSI 0.7 still had an acceptable hop aroma, although the intensity was no longer as strong. This again confirmed that linalool and geraniol are key aroma compounds, since their levels in Styrian Wolf beer were higher than in beers brewed with the other two varieties. However, the intensity and quality were significantly decreased with increasing HSI.

### 3.3. Beer Bitterness

The BU method is most frequently used when the bitterness of beer is in question. However, BU values do not always correlate with the actual perceived bitterness, for two main reasons. The first one is the non-selectivity of the method, as it measures all iso-octane-soluble compounds that absorb at 275 nm. The second one is far more complex, as it concerns the hop variety and hop aroma in terms of perceived bitterness [26]. The HPLC method for the determination of iso-alpha acids and other bittering compounds is more accurate for resolving the first issue. Table 5 shows the results for BU and the alpha acid, iso-alpha acid and humulinone content in beers.

In all three hop varieties, the levels of alpha acids and iso-alpha acids decreased from HSI 0.3 to 0.7. The hop analysis (Table 2) shows that the same holds for the hop samples; therefore, decreased levels are expected in the resulting beer. The isomerisation yield of iso-alpha acids is low for two reasons. One is that the solubility of alpha acids is relatively low, so a greater share remains undissolved, and the other is that the temperatures in dry hopping are not high enough for the thermal isomerisation of alpha acids to iso-alpha acids [24]. The humulinones in all three hop samples (Table 2) increased from HSI 0.3 to 0.7, so the same pattern was expected for the beer. However, the levels of humulinones increased but then began to drop at a certain HSI. The BUs were correlated with the levels of humulinones, meaning that the humulinones in beer were further oxidised into some new compounds that were not detected by the BU method. In 2013, Taniguchi et al. [27] identified 4′-hydroxy-allohumulinones as oxidative products of humulinones that were present only in the hard resin fraction. The hard resin fraction is completely insoluble in hexane; therefore, we suspect that their presence is undetectable using the BU method. The sensorial effect of 4′-hydroxy-allohumulinones on beer bitterness has not been investigated, but the hard resin fraction as a whole has been shown to increase the perceived bitterness and to impart mild bitterness with a well-rounded taste and character [28]. However, distinguishing the sensorial impact of a naturally occurring hard resin fraction and hard resin with oxygenated compounds is important.

In terms of bitterness, the sensorial analysis panellists were asked to evaluate the quality and intensity of beer bitterness from 1 to 5, with half steps allowed. The results are summarised in Table 6. Results are given as the mean value ± standard deviation from ten independent evaluators. The quality of beer bitterness was the highest when hops with the lowest HSI were used. The dynamics for bitterness changes were not equal in all varieties.

**Figure 2:**
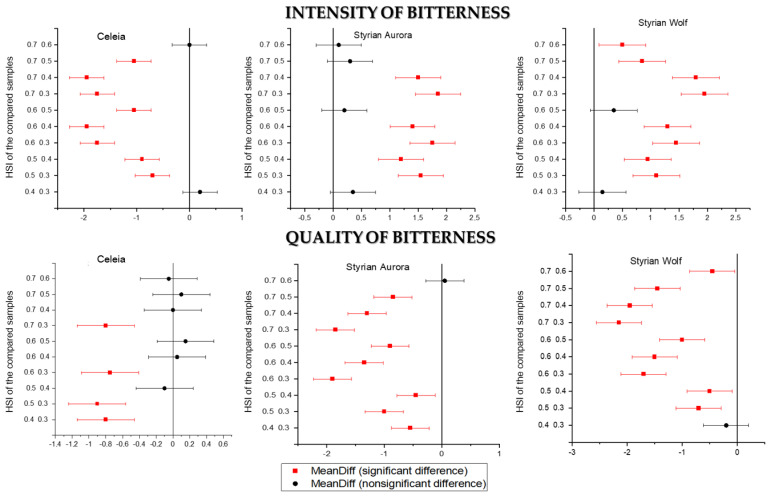
Tukey’s mean comparison plots for intensity and quality of bitterness.

**Figure 3:**
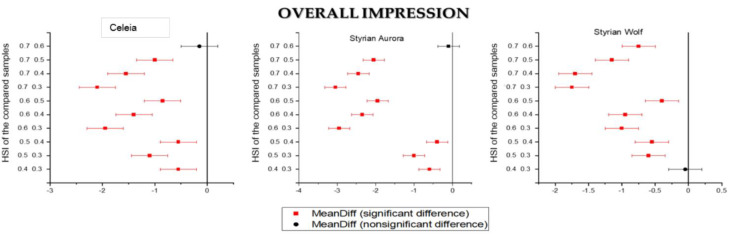
Tukey’s comparison plots for overall impression of beer samples.

Based on the measured bitterness in all three types of beers, after the initial increase, a decrease in the intensity of bitterness was expected. The sensorial intensity of bitterness followed the measured bitterness when Celeia was used for dry hopping. Figure 2 shows the Tukey mean comparison plots for the intensity and quality of the beer samples within an individual variety. No significant difference was noted in the intensity of bitterness between samples 1 and 2 or between samples 4 and 5. Figure 3 shows that the quality of bitterness was significantly higher only when hops with HSI 0.3 were used. Later, the quality showed no significant difference. The perceived bitterness of Aurora beers increased, but no significant differences were noted for samples 8 to 10. Differences were determined in the quality of samples 6 to 9, whereas samples 9 and 10 showed no significant differences. For Styrian Wolf, the intensity of bitterness increased, while the quality slightly decreased.

The quality of bitterness began to drop when hops with HSI values higher than 0.5 were used for dry hopping. An unexpected increase in intensity was observed in beers dry hopped with hops with a high level of alpha acids (Aurora and Styrian Wolf). We assume that an increase in the perceived bitterness comes from oxygenated products stored in the hard fraction of hop resins. Beer bitterness is also affected by hop oil compounds due to synergistic effects between the volatile and non-volatile fractions [29]. Oladokun et al. [26] stated that the bitterness character changes based on the hop aroma profile. Kaltner and Mitter [30] determined that beers with terpene hydrocarbons and low concentrations of linalool have increased bitterness. These findings agree with the results for beers dry hopped with Aurora and Styrian Wolf, but not for Celeia beers. Dietz et al. [21] correlated the presence of geraniol with an increase in smooth bitterness and reported a correlation of alpha-humulene, beta-caryophyllene and humulene epoxides with a harsh and lingering bitterness. This is additional evidence that beer taste is a consequence of numerous factors and cannot be attributed only to one or a few factors.

### 3.4. Overall Impression

Another part of the sensorial evaluation of beer samples was the overall impression. The results are summarised in Table 7, and Figure 3 presents the Tukey comparison plots for overall impression. Samples 1, 6, 11 and 12 achieved the highest grades.

In Aurora and Celeia, there was no statistical difference between samples dry hopped with HSI 0.6 and 0.7, as seen in Figure 3. There was also no difference between samples 11 and 12. All other samples statistically differed. Beers dry hopped with Styrian Wolf possessed a very good overall impression, even at HSI 0.4. Sample 10 (Aurora, HSI 0.7) scored the lowest grade among all samples and sample 11 (Styrian Wolf, HSI 0.3) scored the best for all ten evaluators.

In samples 4, 5, 8, 9, 10, 14 and 15, gushing was detected. This is, to our knowledge, the first evidence that aged hops could induce gushing. In 1973, Gardner and others reported that hop oil is an effective gushing suppressor, especially the hydrocarbon fraction of hop oil, with beta-caryophyllene as the most effective suppressor [31]. Table 3 shows decreases in the concentrations of the hydrocarbon fraction with increasing HSI. Hanke et al. [32] revealed that alpha acids and linalool act as gushing preventers when hops are added late or as dry hops. The oxidation products of alpha acids also tend to increase the gushing potential [33]. The amounts of alpha acids and linalool decrease with ageing, while the levels of oxidation products increase; therefore, our results agree with the available observations made on secondary gushing [31,32,33,34,35]. Generally, beer samples received the highest grades when dry hopped with fresh or slightly aged hops. Overaged hops (HSI > 0.6) do not deliver a well-rounded perception of beer flavour and taste.

## 4. Conclusions

It was shown that old hops with high HSI values affect beer quality. Beer samples dry hopped with higher-HSI hops have decreased levels of hop oil components; therefore, the intensity of the hop aroma is lower. With the increase in oxidation products, the quality of the aroma also begins to decline. In dry hopped beers, the main source of bitterness is humulinones, which are increasing and, at a certain HSI, started to drop; this is due to the further oxidation of humulinones to oxidation products stored in a hard fraction of HSI. Since the quality of bitterness declines with increasing HSI, these oxidation products could have a negative impact on the perceived bitterness of hops. However, further studies are needed to confirm their contribution to the bitterness character of the beer. Hops with HSI 0.3 and lower are the best quality and they impart a well-rounded aroma and good-quality bitterness into beers. There are some changes seen in quality when HSI rises to 0.4. At 0.5, a negative impact is seen mainly in changes in aroma; however, Styrian Wolf is still good for use, if a fresh supply is absent. Hops with HSI over 0.5 are no longer suitable for dry hopping, especially from the perspective of the aroma, which is the main goal of dry hopping techniques. In our study, beer dry hopped with Styrian Wolf with HSI 0.3 was marked as the best from a sensorial point of view. Furthermore, at an HSI of 0.6 in all three varieties, gushing appeared. To our knowledge, we are the first to report that old hops could induce gushing. In conclusion, when a fresh supply is absent, hops with HSI up to 0.4 are acceptable when Celeia and Aurora are used, and up to 0.5 when Styrian Wolf is used.

## Figures and Tables

**Table 1:** Operating conditions for HS-SPME-GS-MS determinations of hop volatiles in beer.

Parameter	Value
SPME fibre	50/30 μm DVB/CAR/PDMS (Supelco, Bellefonte, PA, USA)
Incubation time	7.5 min
Extraction time	20 min
Incubation temperature	60 °C
Agitation rate	500 rpm
Pre-desorption time	20 min
Desorption temperature	250 °C
Liner	Ultra Inert SPME Liner 0.75 mm
Split ratio	1:20
GC column	HP-5MS UI (30 m × 250 μm × 0.25 μm) (Agilent, USA)
Mobile phase	Helium (purity 5.0)
Temperature program	50 °C–190 °C (10 °C/min) 190 °C–300 °C (70 °C/min), 1 min hold
Ion source temperature	230 °C
MS quad temperature	150 °C
Gain	1.000
Acquisition mode	SIM and SCAN

**Table 2:** Content of hop essential oil, alpha acids and humulinones in hop samples, presented as mean value ± standard deviation.

Sample Number	Variety	HSI	Essential Oil [mL/100 g]	Alpha-Acids [%]	Humulinones [%]
1	Celeia	0.3	1.05 ± 0.04	3.19 ± 0.05	0.11 ± 0.01
2	Celeia	0.4	0.89 ± 0.03	2.38 ± 0.03	0.15 ± 0.01
3	Celeia	0.5	0.85 ± 0.03	2.06 ± 0.03	0.17 ± 0.01
4	Celeia	0.6	0.74 ± 0.03	1.80 ± 0.03	0.18 ± 0.01
5	Celeia	0.7	0.46 ± 0.02	1.60 ± 0.02	0.18 ± 0.01
6	Aurora	0.3	1.97 ± 0.08	12.36 ± 0.18	0.43 ± 0.03
7	Aurora	0.4	0.98 ± 0.04	10.38 ± 0.15	0.49 ± 0.03
8	Aurora	0.5	0.79 ± 0.03	8.36 ± 0.12	0.64 ± 0.04
9	Aurora	0.6	0.51 ± 0.02	7.36 ± 0.11	0.65 ± 0.04
10	Aurora	0.7	0.44 ± 0.02	6.05 ± 0.09	0.69 ± 0.04
11	Styrian Wolf	0.3	2.22 ± 0.09	11.87 ± 0.17	0.42 ± 0.03
12	Styrian Wolf	0.4	1.49 ± 0.06	8.66 ± 0.13	0.54 ± 0.03
13	Styrian Wolf	0.5	1.28 ± 0.05	9.21 ± 0.13	0.60 ± 0.04
14	Styrian Wolf	0.6	1.07 ± 0.04	7.71 ± 0.11	0.60 ± 0.04
15	Styrian Wolf	0.7	1.02 ± 0.04	7.66 ± 0.11	0.72 ± 0.05

**Table 3:** Concentrations of hop volatiles in beer samples, presented as mean value ± standard deviation.

Variety	HSI of Hop Used for Beer	Myrcene [μg/L]	Linalool [μg/L]	Alpha-Terpineol [μg/L]	Beta-Citronellol and Nerol [μg/L]	Geraniol [μg/L]	Beta-Caryophyllene [μg/L]	Alpha-Humulene [μg/L]	Caryophyllene-Oxide [μg/L]	Humulene Epoxide [%]	Humulenol II [%]
Celeia	0.3	9588 * ± 144	783 ± 19	115 ± 8	14 ± 1	108 ± 3	993 ± 75	448 ± 70	42 ± 5	0.18 ± 0.01	0.20 ± 0.01
Celeia	0.4	2801 ± 42	699 ± 17	92 ± 7	10 ± 1	90 ± 2	247 ± 19	135 ± 21	76 ± 8	0.36 ± 0.02	0.47 ± 0.03
Celeia	0.5	2018 ± 30	527 ± 13	85 ± 6	28 ± 1	68 ± 2	208 ± 16	121 ± 19	80 ± 8	0.74 ± 0.05	0.66 ± 0.04
Celeia	0.6	1172 ± 18	484 ± 12	86 ± 6	29 ± 1	62 ± 2	136 ± 10	80 ± 13	73 ± 8	0.93 ± 0.06	1.01 ± 0.07
Celeia	0.7	875 ± 13	443 ± 11	81 ± 6	28 ± 1	56 ± 1	53 ± 4	55 ± 9	105 ± 11	1.02 ± 0.07	1.41 ± 0.09
Aurora	0.3	13,721 * ± 206	974 ± 23	124 ± 9	30 ± 1	186 ± 5	606 ± 46	410 ± 64	4 ± 1	0.22 ± 0.01	0.19 ± 0.01
Aurora	0.4	2218 ± 33	594 ± 14	100 ± 7	29 ± 1	125 ± 3	153 ± 12	136 ± 21	23 ± 3	0.48 ± 0.03	0.51 ± 0.03
Aurora	0.5	1327 ± 20	582 ± 14	102 ± 7	10 ± 1	115 ± 3	68 ± 5	66 ± 10	34 ± 4	0.75 ± 0.05	1.25 ± 0.08
Aurora	0.6	639 ± 10	427 ± 10	84 ± 6	28 ± 1	85 ± 2	42 ± 3	44 ± 7	38 ± 4	0.91 ± 0.06	1.92 ± 0.12
Aurora	0.7	332 ± 5	321 ± 8	77 ± 6	25 ± 1	64 ± 2	26 ± 2	27 ± 4	39 ± 4	1.02 ± 0.07	2.52 ± 0.16
Styrian Wolf	0.3	4659 ± 70	1061 ± 25	92 ± 7	31 ± 1	421 ± 11	269 ± 20	162 ± 25	12 ± 1	0.15 ± 0.01	0.16 ± 0.01
Styrian Wolf	0.4	2778 ± 42	944 ± 23	100 ± 7	31 ± 1	337 ± 8	157 ± 12	101 ± 16	28 ± 3	0.35 ± 0.02	0.37 ± 0.02
Styrian Wolf	0.5	2625 ± 40	793 ± 19	99 ± 7	34 ± 1	268 ± 7	133 ± 10	93 ± 15	24 ± 3	0.46 ± 0.03	0.50 ± 0.03
Styrian Wolf	0.6	1678 ± 25	943 ± 23	124 ± 9	47 ± 2	296 ± 7	80 ± 6	55 ± 9	31 ± 3	0.53 ± 0.03	0.95 ± 0.06
Styrian Wolf	0.7	811 ± 12	925 ± 22	155 ± 11	58 ± 3	292 ± 7	60 ± 7	45 ± 7	40 ± 4	0.60 ± 0.04	1.11 ± 0.07

* Outside calibration range.

**Table 4:** Results of sensorial analysis for intensity and quality of beer aroma, presented as mean value ± standard deviation.

Sample Number	Variety	HSI of Hop Used for Beer	Intensity of Hop Aroma	Quality of Hop Aroma
1	Celeia	0.3	4.6 ± 0.32	4.7 ± 0.34
2	Celeia	0.4	3.2 ± 0.48	4.3 ± 0.26
3	Celeia	0.5	2.6 ± 0.44	3.1 ± 0.28
4	Celeia	0.6	1.6 ± 0.21	1.9 ± 0.21
5	Celeia	0.7	1.5 ± 0.24	2.0 ± 0.16
6	Aurora	0.3	4.6 ± 0.32	4.9 ± 0.21
7	Aurora	0.4	3.1 ± 0.21	4.3 ± 0.26
8	Aurora	0.5	2.5 ± 0.28	3.1 ± 0.39
9	Aurora	0.6	1.6 ± 0.32	1.1 ± 0.16
10	Aurora	0.7	1.1 ± 0.21	1.0 ± 0.00
11	Styrian Wolf	0.3	5.0 ± 0.16	5.0 ± 0.16
12	Styrian Wolf	0.4	4.6 ± 0.21	4.8 ± 0.26
13	Styrian Wolf	0.5	4.1 ± 0.28	4.3 ± 0.26
14	Styrian Wolf	0.6	3.0 ± 0.16	3.3 ± 0.35
15	Styrian Wolf	0.7	1.9 ± 0.24	2.8 ± 0.48

**Table 5:** Bitterness units, concentrations of alpha acids, iso-alpha acids and humulinones in beer samples, presented as mean value ± standard deviation. LOD stands for limit of detection.

Sample Number	Variety	HSI of Hop Used for Beer	BU	Alpha Acids [mg/L]	Iso-Alpha Acids [mg/L]	Humulinones [mg/L]
1	Celeia	0.3	24 ± 1	3.60 ± 0.23	0.64 ± 0.05	4.81 ± 0.31
2	Celeia	0.4	28 ± 1	2.71 ± 0.18	0.51 ± 0.04	7.97 ± 0.51
3	Celeia	0.5	23 ± 1	1.78 ± 0.12	0.15 ± 0.01	6.45 ± 0.41
4	Celeia	0.6	22 ± 1	<LOD *	< LOD *	5.19 ± 0.33
5	Celeia	0.7	21 ± 1	<LOD *	< LOD *	3.78 ± 0.24
6	Aurora	0.3	22 ± 1	5.74 ± 0.37	1.52 ± 0.11	16.06 ± 1.03
7	Aurora	0.4	29 ± 1	5.47 ± 0.35	1.46 ± 0.11	19.00 ± 1.22
8	Aurora	0.5	39 ± 2	4.09 ± 0.26	1.27 ± 0.09	21.37 ± 1.37
9	Aurora	0.6	35 ± 1	3.51 ± 0.23	0.85 ± 0.06	19.00 ± 1.22
10	Aurora	0.7	34 ± 1	2.00 ± 0.13	0.42 ± 0.03	17.89 ± 1.14
11	Styrian Wolf	0.3	21 ± 1	4.45 ± 0.29	1.95 ± 0.14	14.03 ± 0.90
12	Styrian Wolf	0.4	25 ± 1	4.05 ± 0.26	1.91 ± 0.14	18.73 ± 1.20
13	Styrian Wolf	0.5	45 ± 2	3.87 ± 0.25	1.19 ± 0.09	30.30 ± 1.94
14	Styrian Wolf	0.6	42 ± 2	2.36 ± 0.15	1.14 ± 0.08	25.26 ± 1.62
15	Styrian Wolf	0.7	35 ± 1	2.94 ± 0.19	0.80 ± 0.06	19.19 ± 1.23

* LOD ˂ 0.1 mg/L.

**Table 6:** Results of sensorial analysis for intensity and quality of bitterness, presented as mean value ± standard deviation.

Sample Number	Variety	HSI of Hop Used for Beer	Intensity of Bitterness	Quality of Bitterness
1	Celeia	0.3	2.9 ± 0.32	4.9 ± 0.21
2	Celeia	0.4	3.1 ± 0.21	4.1 ± 0.32
3	Celeia	0.5	2.2 ± 0.26	4.0 ± 0.24
4	Celeia	0.6	1.2 ± 0.24	4.2 ± 0.24
5	Celeia	0.7	1.2 ± 0.24	4.1 ± 0.32
6	Aurora	0.3	2.4 ± 0.39	4.9 ± 0.21
7	Aurora	0.4	2.8 ± 0.26	4.4 ± 0.24
8	Aurora	0.5	4.0 ± 0.37	3.9 ± 0.21
9	Aurora	0.6	4.2 ± 0.24	3.0 ± 0.33
10	Aurora	0.7	4.3 ± 0.26	3.1 ± 0.28
11	Styrian Wolf	0.3	2.2 ± 0.35	5.0 ± 0.16
12	Styrian Wolf	0.4	2.4 ± 0.34	4.9 ± 0.21
13	Styrian Wolf	0.5	3.3 ± 0.35	4.9 ± 0.24
14	Styrian Wolf	0.6	3.7 ± 0.34	4.4 ± 0.21
15	Styrian Wolf	0.7	4.2 ± 0.24	4.0 ± 0.24

**Table 7:** Results of sensorial analysis for overall impression, presented as mean value ± standard deviation.

Sample Number	Variety	HSI of Hop Used for Beer	Overall Impression
1	Celeia	0.3	4.9 ± 0.21
2	Celeia	0.4	4.4 ± 0.24
3	Celeia	0.5	3.8 ± 0.35
4 *	Celeia	0.6	3.0 ± 0.16
5 *	Celeia	0.7	2.8 ± 0.35
6	Aurora	0.3	4.9 ± 0.21
7	Aurora	0.4	4.3 ± 0.26
8 *	Aurora	0.5	3.9 ± 0.21
9 *	Aurora	0.6	2.0 ± 0.16
10 *	Aurora	0.7	1.9 ± 0.24
11	Styrian Wolf	0.3	5.0 ± 0.00
12	Styrian Wolf	0.4	5.0 ± 0.16
13	Styrian Wolf	0.5	4.4 ± 0.21
14 *	Styrian Wolf	0.6	4.0 ± 0.24
15 *	Styrian Wolf	0.7	3.3 ± 0.26

* Gushing.

## Data Availability

Data is contained within the article.
